
JOURNAL INFORMATION
==============================
Journal ID: Wirtschaftsdienst

Wirtschaftsdienst

EISSN: 1613-978X

ARTICLE INFORMATION
==============================
PMCID: PMC9017739
DOI: 10.1007/s10273-022-3163-y
Article ID: 3163
Article version: 1
Subjects: Zeitgespräch

Energieversorgungsrisiken, Energiepreiskrise und Klimaschutz erfordern gemeinsame Antworten

Energy Supply Risks, the Energy Price Crisis and Climate Protection Require Joint Responses

Fischedick Manfred manfred.fischedick@wupperinst.org

grid.426451.0 0000 0004 0550 8671 Abteilung Energie-, Verkehrs- und Klimapolitik, Wuppertal Institut für Klima, Umwelt, Energie gGmbH, Döppersberg 19, 42103 Wuppertal, Deutschland
Electronic publication date: 2022 Apr 19
Print publication date: 2022
Volume: 102
Issue: 4
First page: 262
Last page: 269
Copyright: © Der/die Autor:in 2022
License: Open Access: Dieser Artikel wird unter der Creative Commons Namensnennung 4.0 International Lizenz veröffentlicht (https://creativecommons.org/licenses/by/4.0/deed.de).
Open Access wird durch die ZBW — Leibniz-Informationszentrum Wirtschaft gefördert.
License URL: https://creativecommons.org/licenses/by/4.0/

Keywords: P18, Q42, Q48
issue-copyright-statement: © The Author(s) 2022

==============================

In den vergangenen zwei Jahren hat sich die Welt verändert und vermehrt beherrschen Krisen das Bild. Jahrzehntelange Gewissheiten gelten nicht mehr, Risiken und Unsicherheiten nehmen zu, die Herausforderungen werden immer komplexer und erfordern gleichzeitig immer schnelleres und konsequenteres Handeln.

Was können und was müssen wir aus den aktuellen Krisen lernen? Wir müssen lernen, dass wir ein erhöhtes Maß an Sensibilität gegenüber potenziellen Risiken brauchen und da, wo notwendig und möglich, Vorsorge treffen müssen und zwar auch dann, wenn dies finanzielle und strukturelle Vorleistungen erfordert. Nach der Coronapandemie ist die zugespitzte geopolitische Lage in Bezug auf die Erdgasversorgung überraschend, aber mit großer Macht auf die Agenda gekommen, das heißt, ohne dass die Entscheidungsträger:innen in Politik und Wirtschaft sowie die Verbraucher:innen darauf wirklich vorbereitet gewesen sind – nicht zuletzt vor dem Hintergrund jahrzehntelanger zuverlässiger Lieferungen (auch zu Zeiten des Kalten Krieges). Was im letzten Quartal des Jahres 2021 mit einem auf verschiedene Effekte[1] zurückzuführenden, drastischen Anstieg der Energiepreise anfing (der Gaspreis ist bei Vertragsverlängerungen oder Neuverträgen für Haushaltsverbraucher:innen im Verlauf des Jahres 2021 um 83 % von 6,47 Cent pro Kilowattstunde auf durchschnittlich 11,84 Cent pro Kilowattstunde gestiegen wie Abbildung 1 zeigt), kumulierte mit dem Krieg Russlands gegen die Ukraine in Bezug auf die Energieversorgung in eine Situation, in der geopolitische Risiken das Bild bestimmen. Hiermit verbunden waren weitere drastische Preisaufschläge beim Energieträger Gas. So stieg der Gaspreis am niederländischen Handelsplatz TTF – dem wichtigsten Index für die EU – am 2. März 2022 von 122 Euro pro Megawattstunde am Vortag auf 166 Euro pro Megawattstunde – also um mehr als ein Drittel an einem Tag. Der zwischenzeitliche Tageshöchststand lag sogar bei 195 Euro pro Megawattstunde, was einem Preisanstieg um knapp 60 % gegenüber dem Vortag entspricht.

Für den Umgang mit der Energieversorgungs- und Energiepreiskrise sind Lösungen und ein dauerhaft tragfähiger Ausweg notwendig. Gleichzeitig ist aber auch klar, dass diese Lösungen nicht zu Lasten der Klimaschutzziele gehen dürfen – um nicht mit einem Beitrag zur Lösung der einen Krise, eine andere, die Menschheit ebenso bedrohende Krise sich weiter zuspitzen zu lassen. Anders ausgedrückt: Die jetzt anstehenden Entscheidungen müssen entsprechend aus einer ganzheitlichen Perspektive getroffen werden. Lock-in-Situationen und Pfadabhängigkeiten sind zu vermeiden, die für das Klima in eine Sackgasse führen.

Umgekehrt ist für die Zukunft vor diesem Hintergrund aber auch klar: Es bedarf eines generellen Risikochecks für die zentralen Energiewende- und Klimaschutzstrategien und -pfade. Dies gilt insbesondere in Bezug auf die Sicherheit der Bereitstellung von Energieträgern, aber für ein Industrieland wie Deutschland sowie in ganz entscheidender Weise für die Versorgung mit Grundstoffen für die Industrie. Hinzu kommt nicht zuletzt die Notwendigkeit einer hinreichenden und sicheren Verfügbarkeit von Rohstoffen, wie etwa Seltene Erden, für die Herstellung zentraler Klimaschutztechnologien oder stabile Bezugsstrukturen für zentrale Komponenten und Produkte. In diesem Sinne gilt es beispielsweise zu überlegen, ob die weitgehende Abhängigkeit vom Import von Photovoltaikmodulen aus China den Anforderungen gerecht wird oder im stärkeren Maße wieder auf eine heimische Produktion gesetzt werden sollte. Dabei geht es nicht darum, vollständig autarke Strukturen aufzubauen, sondern Verletzlichkeiten und Risiken zu reduzieren oder sich dieser

1 Dies betrifft u. a. eine weltweit erhöhte Nachfrage nach Erdgas aufgrund positiver Konjunkturentwicklung, ein kaltes Frühjahr 2021, einen zunehmenden Einsatz von Erdgas in der deutschen Stromversorgung zum Ausgleich des rückläufigen Beitrags erneuerbarer Energien im vergleichsweise sonnen- und windarmen Jahr 2021, einen Anstieg des $CO_2$-Preises, eine zunehmend auch im Jahr 2021 schon angespannte politische Lage sowie zeitweise steigende Stromexporte nach Frankreich aufgrund des Ausfalls eines signifikanten Anteils der französischen Kernkraftwerke.

Abbildung 1

**Entwicklung der Erdgaspreise (Neuverträge) für Haushalte in Mehrfamilienhäusern in Deutschland**

Zahlen für 2022 entsprechen dem Niveau zu Beginn des Jahres

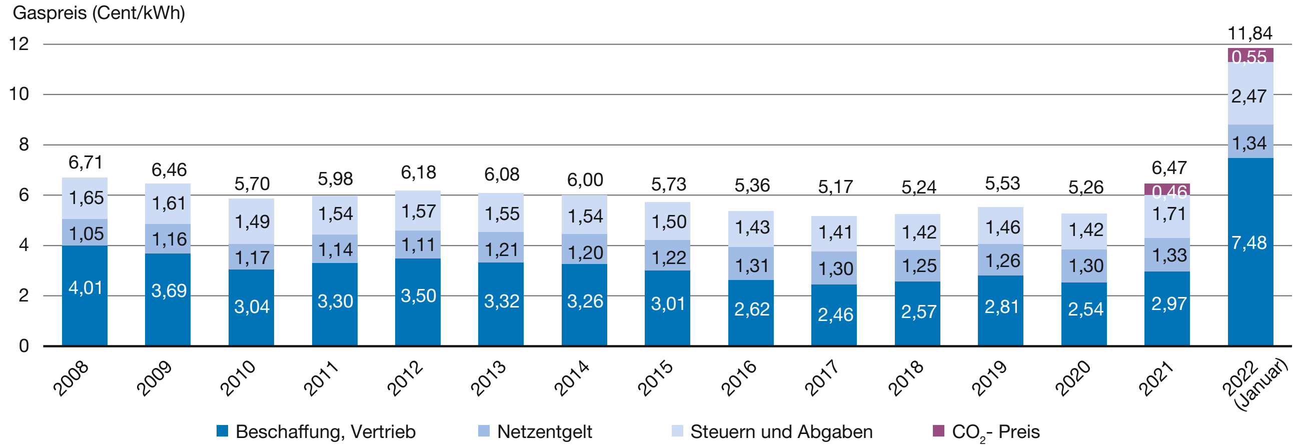

Quellen: BDEW (2021a, 2021b, 2022).

in jedem Fall bewusst zu sein. Neben der Diversifikation von Bezugsstrukturen und einer verstärkten heimischen Produktion kann auch der Übergang der heute nach wie vor stark linear geprägten Produktionsstrukturen auf zirkuläre Strukturen (Circular Economy) einen wesentlichen Beitrag zur Risikominderung leisten.

Vor diesem Hintergrund sollen nachfolgend einige grundlegende Fragestellungen diskutiert werden. Der Schwerpunkt der Fragestellungen liegt dabei auf dem Umgang mit der angespannten Versorgungssituation mit Erdgas.

## Energieversorgungssicherheit erhöhen

In den verschiedenen Sektoren bestehen eine unterschiedliche Abhängigkeit und unterschiedliche Reaktionsfähigkeiten, kurzfristig Alternativen zum Einsatz von Erdgas zu finden. Im Bereich Industrie und Wärmeversorgung von Gebäuden, die beide einen ganz erheblichen Teil der Erdgasnachfrage ausmachen[2] (vgl. Abbildung 2), beziehen sich diese vor allem auf den Übergang zu energieeffizienten Stromanwendungen, Energieeffizienzsteigerungen und ein stärkeres Maß an energiebewusstem Verhalten. Im Bereich der Stromerzeugung besteht neben effizienter Stromnutzung und dem Ausbau der erneuerbaren Energien kurzfristig die Möglichkeit, auf Kohlekraftwerke auszuweichen – verbunden mit dem Nachteil deutlich höherer $CO^2$-Emissionen. Hinzu kommen Möglichkeiten durch Maßnahmen auf der Seite von Gasbeschaffung und Gasbewirtschaftung potenzielle Lücken zu schließen.

Grundsätzlich muss eine Lehre für die Politik aus der kritischen Versorgungsphase sein, die Bemühungen im Bereich Energieeffizienz, vor allem auch Stromeffizienz, die in den vergangenen Jahren eher stiefmütterlich betrachtet worden sind, zu stärken, und den Ausbau erneuerbarer Energien noch einmal deutlich zu forcieren. Denn diese beiden Strategien dienen sowohl dem Klimaschutz als auch der Verringerung unserer Abhängigkeit von Gas, Öl und Kohle. Zudem sind mittlerweile nicht nur viele Energieeffizienzmaßnahmen, sondern auch die erneuerbaren Energien kostengünstiger als neue Energieversorgungsoptionen aus fossiler Energie. Dies gilt auch im Vergleich zu der Errichtung neuer LNG-Terminals.

### Möglichkeiten bei der Wärmeversorgung

Mit Blick auf die kurzfristige Handlungsebene kommt einem in der derzeitigen kritischen Situation sofort die Reaktion in Japan auf die Kernkraftwerksunfälle in Fukushima in den Sinn. Dort ist es durch umfangreiche Maßnahmen gelungen, den Energie- und vor allem den Strombedarf signifikant (zumindest für eine Übergangszeit) zu verringern[3] – allerdings ausgehend von einem sehr hohen Niveau. Dafür hat sich der Begriff Setsuden eingeprägt. Wikipedia (2022) schreibt dazu:

2 Insgesamt betrug der auf Gase entfallene Endenergieverbrauch 2020 im Bereich der Haushalte (primär für die Wärmebereitstellung) 254 Terawattstunden (TWh), im Bereich der Industrie 227 TWh und im Bereich Gewerbe, Handel und Dienstleistungen 98 TWh.

3 In der Region Tokio konnte die Spitzenlast um 10 % bis 15 % gesenkt werden (Lindner, 2017).

Abbildung 2

**Erdgasabsatz und jeweilige Anteile von Erdgas und Erdölgas im Endenergieverbrauch**

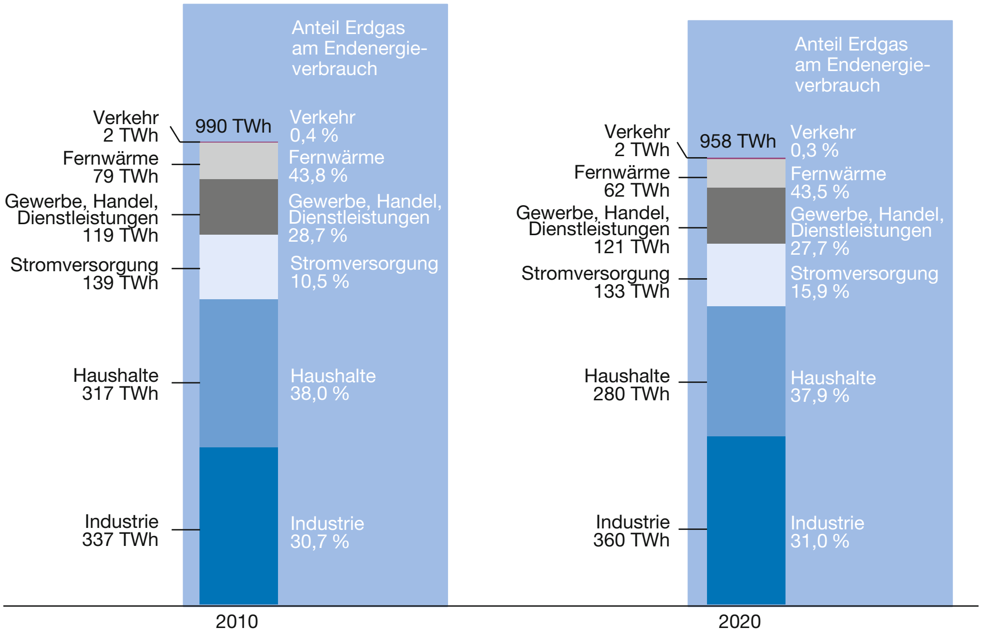

Quellen: eigene Darstellung auf Basis der statistischen Daten von BDEW (2021a), BDEW (2021b) und AGEB (2021).

Setsuden war eine nationale Bewegung in Japan, um die japanische Öffentlichkeit zu ermutigen, in den (traditionell wegen der hohen Klimatisierung verbrauchsintensiven) Sommermonaten 2011 Strom zu sparen und einen insgesamt energiesparenden Lebensstil anzunehmen. Die Bewegung begann im Juli 2011, um Stromausfälle im Sommer aufgrund von Stromknappheit in Ostjapan zu verhindern.

Auch in Deutschland gibt es aktuell einige Möglichkeiten, die schnell zu einer Minderung des Gas- und Ölverbrauchs führen können. In Deutschland ist die Abhängigkeit von Importen aus Russland besonders hoch: Deutschland deckt aktuell etwas mehr als 50 % seines Erdgasverbrauchs über Importe aus Russland, zudem etwa 30 % des Ölbedarfs. Dies betrifft z. B. die Wärmeversorgung in Gebäuden. Energiebewusstes Verhalten, angeregt durch eine breite Informations- und Mitmachkampagne „Intelligentes Heizen und Lüften“ von Bund, Ländern und Kommunen könnte kurzfristig einen Beitrag leisten. Schätzungen zufolge ließen sich im Prinzip 10 % bis 15 % des Heizenergiebedarfs einsparen, wenn wir in den Gebäuden energiebewusster heizen und lüften würden. Denn gerade durch ineffizientes Kipplüften – bei geöffneten Thermostaten – geht viel Energie verloren. Zu energiebewusstem Verhalten gehört auch, auf das Heizen von Räumen zu verzichten, die kaum genutzt werden, sowie auf eine Absenkung der Raumtemperatur zu setzen, die in vielen Haushalten auf ein zu hohes Niveau eingestellt ist – das heißt, dass es hierdurch nicht zu Komforteinbußen kommen muss. Schon eine Reduktion um 1 Grad Celsius würde nach Berechnungen der Internationalen Energieagentur (IEA) europaweit zu einer Reduktion des Gasverbrauchs von rund 10 Mrd. cbm oder umgerechnet 3,5 % führen.

Vermutlich wird zumindest ein Teil der oben genannten Maßnahmen in den Haushalten bereits rein preisgetrieben ohnehin umgesetzt, obwohl wir wissen, dass die Preiselastizität aufgrund vielfältiger Hemmnisse im Gebäudeaber auch im Verkehrssektor eher gering ist. Wie Tabelle 1 zeigt, wird die Preiselastizität im Raumwärmebereich auf -0,2 geschätzt. Das heißt, dass der Energiebedarf bei einer Preiserhöhung um 10 % um etwa 2 % zurückgeht. Entsprechend wird es von der weiteren Entwicklung und Dauerhaftigkeit der Energiepreise abhängen, wie viel sich allein über den Markt regeln wird.

Wie sieht es mit den mittelfristigen Handlungsmöglichkeiten aus? Mit Blick auf den nächsten Winter und danach werden die erwartbaren Preiseffekte allein nicht ausreichen, um die Versorgungssicherheit sicherzustellen. Insofern sind nachfrage- wie angebotsseitig weitere Maßnahmen notwendig. Dies betrifft auf der Nachfra-

Tabelle 1
**Preiselastizitäten der Nachfrage**

| | Anwendungszweck | Energieträger | Preiselastizität |
|---|---|---|---|
| Private Haushalte | Raumwärme | Heizöl leicht | -0,2 |
| | | Erdgas | -0,2 |
| | | Strom | -0,2 |
| | Warmwasser | Heizöl leicht | -0,05 |
| | | Erdgas | -0,05 |
| | | Strom | -0,05 |
| | Elektrogeräte | Strom | -0,025 |
| Gewerbe, Handel, Dienstleistungen (GHD) | Raumwärme | Heizöl leicht | -0,2 |
| | | Erdgas | -0,2 |
| | | Strom | -0,2 |
| Transport und Mobilität | Transport und Mobilität | Benzin | -0,25 |
| | | Diesel | -0,05 |

Quellen: Prognos (2013), auf Basis von Bundesministerium für Wirtschaft und Technologie (2011).

geseite unter anderem ein schnelles und konsequentes Wechseln von Gas zu Strom im Bereich der Wärmeanwendungen – also elektrische Wärmepumpen in Gebäuden. Ähnliche Möglichkeiten ergeben sich auch in der Industrie, die hier nicht detaillierter behandelt werden kann, die aber ebenfalls große Mengen an Gas abnimmt. Elektrodenkessel und Hochtemperatur-Wärmepumpen können hier Gasanwendungen ersetzen.

- eine Sanierungsoffensive im Gebäudebestand in Abhängigkeit und der zeitlichen Reihenfolge der Wärmedämmstandards der Gebäude. Individuelle Sanierungsfahrpläne werden hierbei eine zentrale Rolle spielen müssen. Wärmedämmung spart zudem auch Kosten bei der Umstellung auf Wärmepumpen, denn sie ermöglicht, mit den niedrigeren Temperaturen der Wärmepumpenheizung die Gebäude zu erwärmen. Sonst müssten oft größere Heizkörper oder eine Fußbodenheizung eingebaut werden.

- eine Ausweitung der Nah- und Fernwärmeversorgung, die parallel von fossilen auf erneuerbare Energien – also auf Solarthermie, Geothermie, Großwärmepumpen, beziehungsweise industrielle Abwärme – umgestellt werden muss.

Eine derartige Entwicklung ist kein Selbstgänger, sondern erfordert ein mutiges Konzept des „Forderns und Förderns", rasches und konsequentes Handeln der betroffenen Akteur:innen und eine geschlossene gesellschaftliche Unterstützung. Hinzu kommt eine Konzentration verfügbarer Kapazitäten (unter anderem im Handwerk) in strategisch relevanten Bereichen (z. B. mehr Sanierungsmaßnahmen statt Verschönerungsmaßnahmen, die für eine gewisse Zeit warten können) und die Flankierung mit unterstützenden Prozessen, wie etwa Qualifizierungs- und Ausbildungsoffensive. Dienstleistungsangebote wie One-Stop-Shops, die Komplettangebote für Gebäudesanierung machen und die schnelle Einführung innovativer Technologien wie das serielle Sanieren sind weitere wichtige Grundvoraussetzungen für die substanzielle Rückführung des Energiebedarfs in Gebäuden.

Nachfolgende Aufzählung beschreibt ein Sechs-Punkte-Sofortprogramm für die Wärmewende (Thomas et al. 2022), mit dem es möglich erscheint, in die beschleunigte Umsetzung zu gehen und schon bis 2035 die Wärmeversorgung der Gebäude in Deutschland vollständig auf erneuerbare Energien umzustellen:

1. Ein Ausstiegsgesetz für Öl- und Gasheizungen. Die Autoren schlagen vor, bis 2024 ein Verbot des Neueinbaus von Erdgas- und Ölheizungen zu erlassen sowie einen Stufenplan für ein Betriebsverbot einzuführen: ab 2027 für alle Anlagen, die vor 2000 eingebaut wurden, ab 2030 für alle Anlagen, die vor 2010 eingebaut wurden und dann für alle verbleibenden bis 2035.

2. Ein Förderprogramm für zukunftsfähige Gebäudewärme in Einzelanlagen mit dem Ziel, bis 2035 12 Mio. neue Wärmepumpen zu verbauen und eine Gesamtfläche von Solarthermieanlagen von 70 Mio. Quadratmetern zu erreichen.

3. Eine Pflicht zur energetischen Sanierung für ineffiziente Gebäude mit ökologischen Kriterien. Dazu schlagen die Autoren vor, dass alle Gebäude in den Effizienzklassen E und H bis 2030 die Klasse D erreicht haben müssen, danach alle Gebäude bis 2035 die Klasse C und bis 2040 die Klasse B. Dabei unterstützen soll der individuelle Sanierungsfahrplan. Allein der Einbau einer Wärmepumpe ermögliche nach aktuellem Recht bereits eine Verbesserung um zwei bis drei Effizienzklassen.

4. Ein Förderprogramm zur energetischen Sanierung von jährlich mindestens 3 % des Gebäudebestands mit ökologischen Kriterien.

5. Ein Erneuerbare-Wärmenetze-Gesetz. Dieses soll festschreiben, dass das Wärmenetz bis 2035 vollständig dekarbonisiert ist, dass leitungsgebundene Wärme ausgebaut wird und die Entwicklung hin zu Niedertemperatur-Wärmenetzen geht.

6. Ein Förderprogramm für zukunftsfähige Wärmenetze. Dieses soll entsprechend der Ausbauziele im Erneuerbare-Wärmenetze-Gesetz gestaltet werden und unter anderem den Umbau auf Niedertemperatur fördern sowie „grüne“ Wärmeerzeugung und -einspeisung.

## Möglichkeiten im Stromsektor

Welche Möglichkeiten ergeben sich im Stromsektor, in dem heute auch viel Gas zum Einsatz kommt[4] und in dem Gas bisher als wichtige Brückentechnologie und komplementäre Stromerzeugungsoption zum Ausgleich des schwankenden Angebots von Strom aus Wind- und Sonnenenergie verstanden wird?

Die in Deutschland installierten Stein- und Braunkohlekraftwerke sind derzeit nicht vollständig ausgelastet. Zudem befindet sich eine Vielzahl von Kraftwerken in der sogenannten Reservehaltung. Hierdurch ergibt sich eine kurzfristige Möglichkeit, die Verstromung von Erdgas zu ersetzen, sofern die Erdgaskraftwerke nicht zur Versorgung von Fernwärmesystemen zum Einsatz kommen. Dass diese Möglichkeit nicht rein theoretischer Natur ist, zeigen die Entwicklungen im Jahr 2021: In dem Jahr hat sich der Anteil der Kohle an der Stromerzeugung gegenüber dem Vorjahr sehr deutlich im Zuge des Anstiegs der Erdgaspreise erhöht – trotz der hohen $CO_2$-Preise. Verbunden war diese Entwicklung mit dem Nachteil deutlich höherer $CO_2$-Emissionen. Diese Verlagerung im Stromerzeugungsmix wird sich 2022 fortsetzen. Hintergrund sind dabei vorrangig die Preiseffekte am Markt und weniger eine etwaige physische Verknappung von Erdgas.

Inwieweit die Verschiebung in der Stromerzeugungsstruktur anhält, hängt von verschiedenen Faktoren ab. Aus Sicht des Klimaschutzes ist sie nicht gewünscht. Insofern ist gut zu wissen, dass sich mittel- und längerfristig eine ganze Reihe von Möglichkeiten ergibt, Kohle durch klimaverträglichere Optionen zu ersetzen und damit auch an dem von der Bundesregierung für 2030 geplanten Kohleausstieg festhalten zu können. Dies wird aber nur dann gelingen, wenn substanzielle Maßnahmen in folgenden Bereichen greifen:

- Drastisch beschleunigter Ausbau erneuerbarer Energien für die Stromerzeugung (unter anderem durch eine deutliche Verringerung der Planungs- und Genehmigungszeiten und eine gegenüber heute breitere gesellschaftliche Akzeptanz). Die verstärkte endenergieseitige Umstellung auf Stromanwendungen (Erhöhung der Flexibilität durch Sektorenkopplung) kann dabei im Verbund mit Speichersystemen helfen, die Fluktuationen der Stromerzeugung aus erneuerbaren Energien abzupuffern.
- Eine Umstellung von kohle- aber auch erdgasbasierten Heizkraftwerken auf grüne Fernwärme (durch die Einbindung von solarthermischen Kollektorsystemen, Geothermie, industrielle Abwärme, thermische Müllverwertungsanlagen, Wärmepumpen).
- Flankierend dazu eine Energie-/Stromeffizienzoffensive, die bei den traditionellen Stromanwendungen, wie bei „weißer Ware“ (elektrische (Küchen-)Geräte) in Haushalten, Motoren und Antrieben wie Pumpen in der Industrie, zu einer verringerten Nachfrage nach Strom führt.

Schnellerer Ausbau erneuerbarer Energien (vor allem Photovoltaik und Windenergie) sowie verstärkte Energie- und Stromeffizienzbemühungen sind jetzt das Gebot der Stunde, damit der negative Klimaeffekt nicht zu groß wird. Im Unterschied dazu wird die Kernenergie in Deutschland keinen sinnvollen Beitrag zur Substitution der Erdgasverstromung leisten können. Die noch laufenden drei Kernkraftwerke sind (in ihren Wartungszyklen und der Brennstoffbevorratung) seit langem auf ein Abschalten Ende 2022 ausgelegt und müssten entsprechend ertüchtigt werden, um bei annehmbaren Sicherheitsbedingungen länger laufen zu können, was wiederum Geld aber auch Zeit kostet. Neue Kernkraftwerke sind in Deutschland aus Gründen der gesellschaftlichen Akzeptanz nicht umsetzbar. Sie würden aufgrund der langen Planungs- und Genehmigungszeiten aber ohnehin deutlich zu spät kommen und wären – wie die gerade in der Umsetzung befindlichen Projekte in Frankreich, Finnland und Großbritannien zeigen – auch energiewirtschaftlich aufgrund der hohen Kosten nicht sinnvoll.

## Möglichkeiten auf der Seite der Gasbeschaffung

Begleitet werden müssen die nachfrageseitigen Maßnahmen durch Veränderungen auf der Angebotsseite wie unter anderem:

- eine Überprüfung der Möglichkeiten, um die heimische Gasförderung zu erhöhen (derzeit kann Deutschland nur rund 5,2 % des eigenen Bedarfs durch eigene Vorkommen decken) unter Berücksichtigung der geologischen Potenziale aber auch der ökologischen Grenzen, das heißt, kein Einstieg in Fracking-Gas.
- eine weitere Diversifizierung der Gasimportströme (soweit möglich Erhöhung des Bezugs aus den Nie-

4 Im Jahr 2020 waren es 171 Terawattstunden.

derlanden[5] und Norwegen, Aufbau und Ausweitung von LNG-Bezugs- und Verarbeitungsstrukturen durch eigene Terminals mit der Möglichkeit, diese auf Wasserstoff umzustellen, um einen fossilen Lock-in zu vermeiden[6], sowie den Bezug von LNG aus Ländern wie den USA, Ägypten und Katar.

- ein konsequenteres Gasspeichermanagement (mit der Vorgabe von Mindestfüllständen in kritischen Zeiten).
- einen deutlich beschleunigten Aufbau einer grünen Wasserstoffinfrastruktur, um spätestens nach 2030 (besser früher) fossiles Gas durch grünen Wasserstoff sukzessive zu ersetzen. Längerfristig kann grüner Wasserstoff Erdgas als eine zentrale Säule der Energieversorgung ablösen. Beim Aufbau der Wasserstoffstrukturen ist dabei – den aktuellen Lehren folgend – noch stärker als bisher auf ein hohes Maß an Diversifizierung der Wasserstoff-Importquellen und hohe Flexibilität zu achten, um in keine neuerlichen einseitigen Abhängigkeiten zu geraten. Zudem sollte der Aufbau heimischer Erzeugungskapazitäten gestärkt werden und ein starker Fokus auf europäische Zusammenarbeit gelegt werden. Im internationalen Kontext bedarf es des Aufbaus von vertrauensvollen Energie- und Wasserstoffpartnerschaften, die die Eigeninteressen der Exportländer bei der Entwicklung ihrer Energiesysteme auf Augenhöhe berücksichtigen.
- eine konsequente Vermeidung von Lock-in durch die Umsetzung von Ersatzmaßnahmen für die Substitution von russischem Erdgas unter anderem durch die Sicherstellung der Umwandlungsfähigkeit neuer Erdgasstrukturen auf Wasserstoff ($H_2$-Readiness).

Unter diesen Bedingungen verbleibt vermutlich hinreichend viel fossiles Gas im System, um dieses auch im Jahr 2030 in den Bereichen zum Einsatz bringen zu können, in denen Substitutionsmöglichkeiten in dieser Dekade begrenzt sind.

Der Angriff Russlands auf die Ukraine hat aber auch gezeigt, dass es zukünftig wichtiger wird, bei der Gestaltung des Energiesystems der Zukunft bereit zu sein, die Verletzlichkeiten transparent zu machen. Dies schließt ein, im Rahmen von “Black Swan”-Analysen auch das Undenkbare in seinen Konsequenzen zu durchdenken sowie sich der bestehenden täglichen Risiken wie etwa durch den Betrieb von Kernkraftwerken bewusst zu sein.

## Energiewende und Klimaschutz

Werden die oben genannten Maßnahmen umgesetzt, ergeben sich – zumindest kurzfristig – teilweise gegensätzliche Wirkungen in Bezug auf den Klimaschutz. Während sich die Umsetzung von Energieeffizienzmaßnahmen und energiebewusstes Verhalten direkt positiv auswirken, führt der teilweise Umstieg von der Gas- auf die Kohleverstromung zu höheren Treibhausgasemissionen. Dies gilt gegenüber dem gegenwärtigen Status auch für den Ersatz von russischem Erdgas durch LNG, das aufgrund der Umwandlungsverluste (von der gasförmigen in die flüssige Phase und zurück) aber auch aufgrund der potenziellen Quellenstruktur (insbesondere wenn es sich um LNG aus den USA mit signifikanten Anteilen an Fracking-Gas handelt) höhere Emissionen aufweist.

Über diese direkten Wirkungen hinaus sind auch systemische Wirkungen zu beachten. Im Bereich der Energieanwendungen besteht die Problematik, dass viele Maßnahmen zwar den Druck aus der Gasversorgung nehmen beziehungsweise diesen zumindest verringern können, manche aber zu einem erhöhten Strombedarf führen, wie etwa elektrische Wärmepumpen in den Haushalten, Elektrodenkessel in der Industrie. Geht man davon aus, dass der Strom zumindest für eine Übergangszeit mit einem höheren Anteil in Kohlekraftwerken erzeugt würde, wirkt dies auf den ersten Blick aus Sicht des nationalen Klimaschutzes kontraproduktiv. Bei unveränderten Mengenbegrenzungen im europäischen Emissionshandelssystem würden die Effekte insgesamt grenzüberschreitend ausgeglichen. Auf den zweiten Blick wird aber schnell klar, dass es zu diesem Vorgehen in einer für die sukzessive Marktdurchdringung notwendigen Hochlaufphase der Stromanwendungen keine Alternative gibt, sondern damit Vorleistungen erbracht werden für einen dauerhaft positiven Klimaschutzeffekt in einem dann zukünftig weitgehend auf erneuerbaren Energien basierenden Stromsystem.

Umso wichtiger ist, dass dieser negative Klimaschutzeffekt auf der Zeitachse durch ein höheres Dekarbonisierungstempo im Zuge des Ausbaus erneuerbarer Energien ausgeglichen werden muss. Denn für den Klimaschutz zählen nicht die Treibhausgasemissionen an einem bestimmten Stichtag, sondern die kumulierten Emissionen. Höhere Emissionen in einer früheren Phase des Transformationspfades können entsprechend durch

5 Nach aktuellen Planungen streben die Niederland an, die Erdgasförderung aufgrund der Erdbebengefahr im Jahr 2029 zu beenden. Hinzu kommt die bereits in der Umsetzung befindliche Umstellung der Versorgung in Deutschland auf H-Gas und damit eine Abkehr vom niederländische L-Gas. Vor diesem Hintergrund stellt ein vermehrter Import aus den Niederlanden vermutlich lediglich eine kurz- bis mittelfristige Option dar.

6 Die Bundesregierung hat Anfang März 2022 entschieden, dass zwei LNG Terminals (in Wilhelmshaven und Brunsbüttel) mit Unterstützung des Bundes gebaut und zu Mitte des Jahrzehnts ihren Betrieb aufnehmen sollen.

Abbildung 3

**Status quo der Importe im Vergleich zu der Entwicklung im Rahmen der Umsetzung klimaverträglicher Transformationspfade**

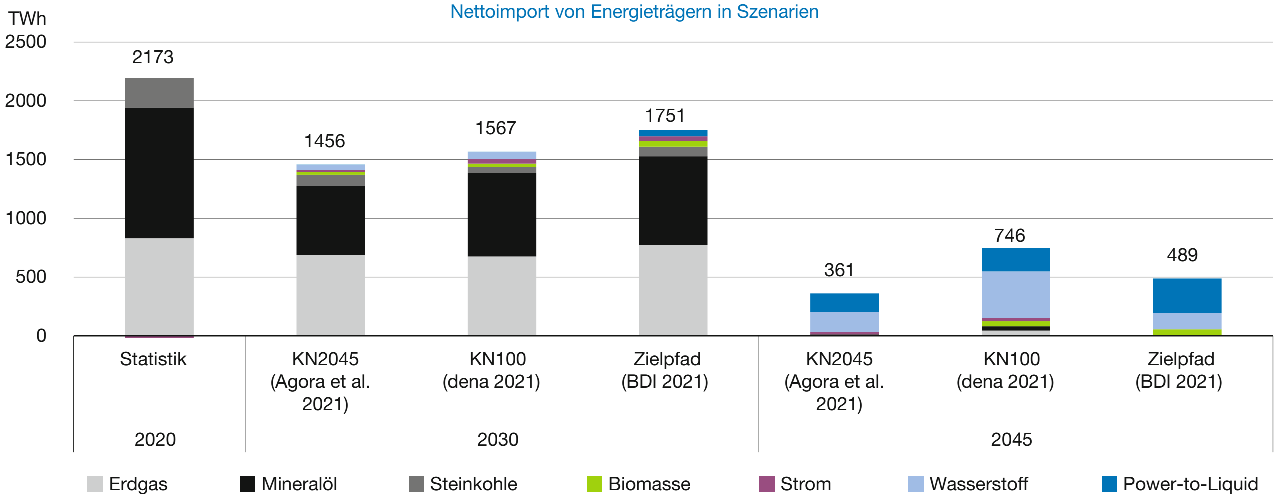

Quellen: eigene Darstellung auf Basis der statistischen Daten für das Jahr 2020 der AGEB (2021).

geringere Emissionen zu einem späteren Zeitraum ausgeglichen werden. Dabei ist allerdings zu berücksichtigen, dass die Bundesregierung mit dem Koalitionsvertrag das Ausbauziel der erneuerbaren Energien für das Jahr 2030 bereits von 65 % auf 80 % (Anteil am Stromverbrauch) erhöht hat.

Die zentrale Herausforderung ist damit der beschleunigte Ausbau erneuerbarer Energien, vor allem im Bereich der Stromerzeugung, aber auch bei den Wärmeanwendungen. Damit stellt sich die Kernfrage, ob es angesichts der verschärften Herausforderungen (Klimaschutz und Energieversorgungssicherheit) jetzt gelingt, einen breiten gesellschaftlichen und politischen Konsens zu erzeugen inklusive der Bereitschaft aller Beteiligten, und gemeinsam pragmatische Lösungen zu finden und Partialinteressen zugunsten des Gemeinwohls zurückzustellen. Dies schließt die Überwindung des sogenannten NIMBY-Effektes („Not In My Backyard“) ebenso mit ein wie den Abbau von Restriktionen, die sich aus bisher ungelösten Konflikten (z. B. Vogel- und Landschaftsschutz versus Ausbau der Windenergie) ergeben, und die drastische Vereinfachung des Genehmigungsrechts und der damit verbundenen Gefahren sowie den deutlichen Ausbau der Bearbeitungskapazitäten in den Genehmigungsbehörden. Gelingt es, diese Hemmnisse gegebenenfalls auch durch eine deutlich proaktivere Rolle des Staates – etwa bei der Errichtung von neuen Infrastrukturen – zu überwinden und ohne Partizipationsmöglichkeiten grundsätzlich einzuschränken, kann dies der Schlüssel für eine sogar beschleunigte Energiewende sein. Wenn nicht jetzt, wann dann sollten die Ausgangsvoraussetzungen dafür gegeben sein, den Mut für neue und schnell umsetzbare Lösungen aufzubringen?

Gelingt es, diesen Weg zu gehen, kann nicht nur ein Beitrag für den Klimaschutz geleistet werden, sondern auch die Importabhängigkeit Deutschlands deutlich verringert werden. Während heute noch mehr als 70 % der Primärenergieträger importiert werden müssen, zeigen die aktuell für Deutschland vorliegenden Transformationsstudien (diese Studien skizzieren Pfade, mit denen Deutschland bis 2045 treibhausgasneutral werden kann[7] wie Abbildung 3 verdeutlicht), dass sich die Importabhängigkeit stark verringern kann und je nach Szenario bis 2045 auf nur noch 20 % bis 42 % zurückgehen wird. Dabei müssten künftig vor allem Wasserstoff, aus Wasserstoff abgeleitete synthetische Kraft- und Brennstoffe (Power-to-X, PtX), Strom und eventuell Biomasse importiert werden. Deutschland kann damit künftig nicht autark werden, die Energieversorgungssicherheit ließe sich unter der Voraussetzung diversifizierter Importstrukturen aber deutlich besser ausgestalten als heute – wo vor allem Öl, Gas und Steinkohle sowie Uranbrennstoffe importiert werden, mit

7 Entsprechende Studien sind im Jahr 2021 im Auftrag von Agora Energiewende, der Deutschen Energieagentur (DENA), dem Bundesverband der Deutschen Industrie (BDI) und dem Bundeswirtschaftsministerium (BMWi) erstellt worden. Letztere allerdings mit der veralteten Zielsetzung der Erreichung von Treibhausgasneutralität im Jahr 2050 (die Szenarien sind in den Vergleich nicht aufgenommen worden).

zum Teil hohen einseitigen Abhängigkeiten von einzelnen Staaten wie Russland.

## Ausblick

Die Energieversorgungs- und Energiepreiskrise kann dementsprechend durchaus beschleunigend auf die Umsetzung der Energiewende und insbesondere der Klimaschutzziele wirken – gleichsam als „Booster“. Dies gilt vor allem dann, wenn die Botschaft verstanden wird, dass Krisenprävention aufgrund der gemachten Erfahrungen einen deutlich höheren Stellenwert bekommen muss. Dies schließt die Begrenzung der Folgen des Klimawandels explizit mit ein. Mit der Energieversorgungs- und Energiepreiskrise werden die Gründe dafür, schnell und konsequent umzusteuern, noch vielfältiger. Hohe Energieträgerpreise, potenzielle Energieträgerknappheiten, aber auch zunehmende Wetterextreme weltweit und in Deutschland – wie die Ereignisse im Juli 2021 gezeigt haben –, sind gute Gründe jetzt zu handeln.

In the past two years, the world has changed and global crises (e. g. the COVID-19 pandemic, war in Ukraine) increasingly dominate the headline. Decades of certainties no longer apply, risks and uncertainties are increasing, challenges are becoming more complex and at the same time require faster and more consistent action. Against that background, the text discusses what we can — and must — learn from the current crises. We must learn that we need to be more sensitive to potential risks and take precautions where necessary and possible, even if this requires pre-emptive financial and structural actions. The text focuses onis the current situation in which we have to find integrative answers reflecting the severe energy supply risks (associated with the energy import dependency from Russia) and the rising and volatile energy prices on the one hand and the climate protection challenge on the other.

Prof. Dr.-Ing. Manfred Fischedick ist Wissenschaftlicher Geschäftsführer des Wuppertaler Instituts für Klima, Umwelt, Energie.


Literatur

AGEB — AG Energiebilanzen e. V. (Hrsg.) (2021), Auswertungstabellen zur Energiebilanz Deutschland Daten für die Jahre von 1990 bis 2020 — Stand: September 2021 (endgültige Ergebnisse bis 2019, vorläufige Daten für 2020).
BDEW — Bundesverband der Energie- und Wasserwirtschaft e. V. (2022), BDEW-Gaspreisanalyse Januar 2022, https://www.bdew.de/service/daten-und-grafiken/bdew-gaspreisanalyse/ (7. März 2022).
BDEW — Bundesverband der Energie- und Wasserwirtschaft e. V. (2021a), Erdgasabsatz in Deutschland Erdgasabsatz in Deutschland nach Verbrauchergruppen — Zehnjahresvergleich [Diagramm], https://www.bdew.de/media/documents/Erdgasabsatz_nach_Kundengruppen_Vg_10J_o_jaehrlich_Ki_online_03052021.pdf (14. März 2022).
BDEW — Bundesverband der Energie- und Wasserwirtschaft e. V. (2021b), Entwicklung des Erdgasabsatzes in Deutschland, https://www.bdew.de/service/daten-und-grafiken/entwicklung-des-erdgasabsatzes-deutschland/ (9. März 2022).
Bundesministerium für Wirtschaft und Technologie (BMWi) (Hrsg.) (2011), 2. Nationaler Energieeffizienz-Aktionsplan (NEEAP) der Bundesrepublik Deutschland — Gemäß EU-Richtlinie über Endenergieeffizienz und Energiedienstleistungen (2006/32/EG) sowie Gesetz über Energiedienstleistungen und andere Energieeffizienzmaßnahmen (EDL-G) — Methodisches Begleitdokument, https://www.buildup.eu/sites/default/files/content/DE%20-%20Energy%20Efficiency%20Action%20Plan%20Technical%20DE.pdf (14. März 2022).
Lindner, R. (2017), Setsuden: Die Energiekrise und gesellschaftliche Stromsparanstrengungen nach Fukushima, in I. Wieczorek und D. Chiavacci (Hrsg.), Japan 2017, 201–216, http://vsjf.net/wp-content/uploads/2020/03/jjb_2014_09_lindner.pdf (14. März 2022).
Prognos AG (Hrsg.) (2013), Endenergieeinsparziel gem. Art. 7 EED und Abschätzung der durch politische Maßnahmen erreichbaren Energie-einsparungen, im Auftrag der BfEE, https://www.bmwi.de/Redaktion/DE/Publikationen/Studien/endenergieeinsparziel-abschaetzungder-durch-politische-massnahmen-erreichbaren-energieeinsparungen.pdf?__blob=publicationFile&v=5 (14. März 2022).
Thomas, S., D. Schüwer, F. Vondung und O. Wagner (2022), Heizen ohne Öl und Gas bis 2035 — ein Sofortprogramm für erneuerbare Wärme und effiziente Gebäude, Studie im Auftrag von Greenpeace e. V., Wuppertal.
Wikipedia (2022), Setsuden, https://en.wikipedia.org/wiki/Setsuden (23. März 2022).
